# The Proteasome Distinguishes between Heterotypic and Homotypic Lysine-11-Linked Polyubiquitin Chains

**DOI:** 10.1016/j.celrep.2015.06.061

**Published:** 2015-07-16

**Authors:** Guinevere L. Grice, Ian T. Lobb, Michael P. Weekes, Steven P. Gygi, Robin Antrobus, James A. Nathan

**Affiliations:** 1 Cambridge Institute for Medical Research, Department of Medicine, University of Cambridge, Cambridge Biomedical Research Centre, Cambridge CB2 0XY, UK; 2 Department of Cell Biology, Harvard Medical School, Boston, MA 02115, USA

## Abstract

Proteasome-mediated degradation occurs with proteins principally modified with lysine-48 polyubiquitin chains. Whether the proteasome also can bind atypical ubiquitin chains, including those linked by lysine-11, has not been well established. This is critically important, as lysine-11 polyubiquitination has been implicated in both proteasome-mediated degradation and non-degradative outcomes. Here we demonstrate that pure homotypic lysine-11-linked chains do not bind strongly to the mammalian proteasome. By contrast, heterotypic polyubiquitin chains, containing lysine-11 and lysine-48 linkages, not only bind to the proteasome but also stimulate the proteasomal degradation of the cell-cycle regulator cyclin B1. Thus, while heterotypic lysine-11-linked chains facilitate proteasomal degradation, homotypic lysine-11 linkages adopt conformations that prevent association with the proteasome. Our data demonstrate the capacity of the proteasome to bind ubiquitin chains of distinct topology, with implications for the recognition and diverse biological functions of mixed ubiquitin chains.

## Highlights

• Homotypic K11-polyubiquitin chains do not bind strongly to the proteasome
• Proteasome shuttling factors preferentially bind K48 chains compared to K11 chains
• Heterotypic but not homotypic K11 chains stimulate proteasomal degradation
• The proteasome disassembles both homotypic and heterotypic K11-polyubiquitin chains

## Graphical Abstract

**Figure:**
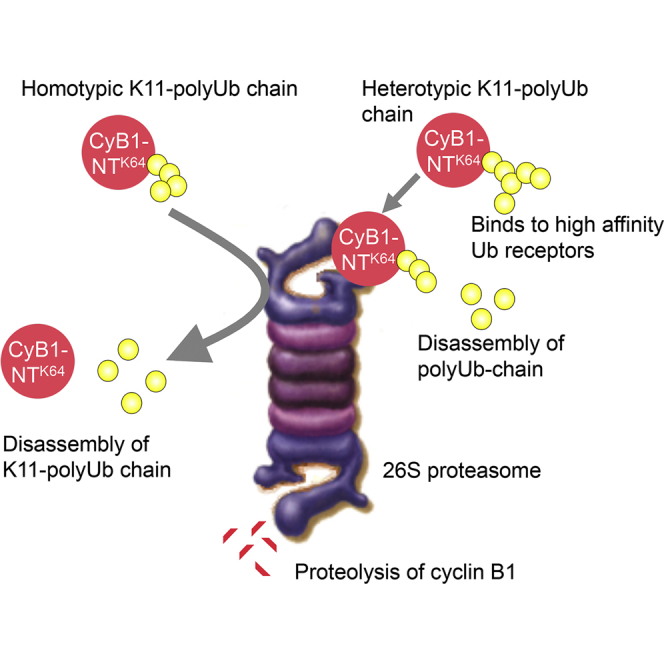

## Teaser

It is unclear whether atypical polyubiquitin chains signal proteasomal degradation. Grice et al. show that, while heterotypic lysine-11-linked polyubiquitin chains bind to the proteasome and stimulate degradation of cyclin B1, proteins modified with homotypic lysine-11-linked chains neither bind strongly to proteasomes nor signal efficient degradation.

## Introduction

Complexity and specificity in the ubiquitin (Ub) proteasome system is generated by the ability of Ub to form eight different chain linkages on itself, through its seven lysine residues (K6, K11, K27, K29, K33, K48, and K63) or N terminus. Each type of Ub linkage is thought to adopt a unique topology and can form a distinct binding surface. Further complexity is generated by the formation of polyubiquitin (polyUb) chains, which can be through the same Ub linkage (homotypic chains) or a combination of different lysine linkages, resulting in mixed or branched structures (heterotypic chains). It is only through recognition of these different Ub structures by Ub-binding proteins (UBPs) that the intracellular fate of the protein is determined.

All polyubiquitin chains so far tested can bind directly to the proteasome in vitro, the most well-studied and abundant linkages being those of K48 and K63. K48-polyUb chains signal proteasomal degradation, and they are targeted for degradation by the 26S proteasome through association with the Rpn10 and Rpn13 receptors of the regulatory 19S particle (Husnjak et al., 2008; Schreiner et al., 2008). Although K63-polyUb chains also bind to the proteasome with similar affinity in vitro, in cells K63 chain binding to the proteasome is blocked by K63-specific UBPs (Nathan et al., 2013). K63-polyUb chains instead mediate protein function in intracellular signaling, DNA repair, and endosomal-lysosomal degradation (Ikeda and Dikic, 2008).

The functions of other Ub linkages are only beginning to emerge, but K11-linked polyUb chains are of particular interest given their abundance (Xu et al., 2009), unique chain structure (Bremm et al., 2010; Castañeda et al., 2013), and association with cell-cycle progression (Jin et al., 2008; Wu et al., 2010). It is also unclear whether K11-Ub chains are a direct signal for proteasomal degradation or mediate other intracellular pathways. In support of a role for K11-polyUb in promoting proteasomal degradation, the anaphase-promoting complex/cyclosome (APC/C) recruits the K11-specific E2 enzyme, Ube2S, to form K11-polyUb chains on several cell-cycle regulators, including cyclin B1 and securin (Garnett et al., 2009; Jin et al., 2008; Wu et al., 2010), the proteasomal-mediated degradation of which promotes mitotic exit. However, the absolute requirement for the formation of K11-polyUb linkages is controversial, as multiple monoubiquitin (monoUb) linkages are sufficient for mitotic progression (Dimova et al., 2012), and chains formed by the APC/C contain heterotypic branched K48 and K11 linkages, rather than homotypic K11 chains (Meyer and Rape, 2014). Furthermore, K11-Ub chains on other proteins also have proteasome-independent functions, including intracellular signaling (Bremm et al., 2014; Dynek et al., 2010), endocytosis (Boname et al., 2010), and even stabilization of K11-polyUb substrates (Dao et al., 2012; Qin et al., 2014). Thus, there is a fundamental need to understand how K11 linkages are recognized in cells and whether K11-polyUb chains bind directly to the proteasome to trigger degradation.

Here we show that homotypic K11-polyUb conjugates do not bind significantly to isolated mammalian 26S proteasomes, the 19S regulatory particle, or the Rad23 proteins, and that free K11-linked chains cannot compete with K48-linked chains for binding to the 19S Ub receptors. However, heterotypic K11/K48-polyUb chains bind to the proteasome and facilitate the degradation of cyclin B1. Homotypic K11-polyUb chains, therefore, adopt a unique topology that prevents their association to the proteasome, which highlights a unique capacity of Ub receptors to discriminate between homotypic and heterotypic polyUb-chain structure.

## Results

### Homotypic K11-PolyUb Chains Do Not Bind Strongly to the 26S Proteasome

**Figure 1:**
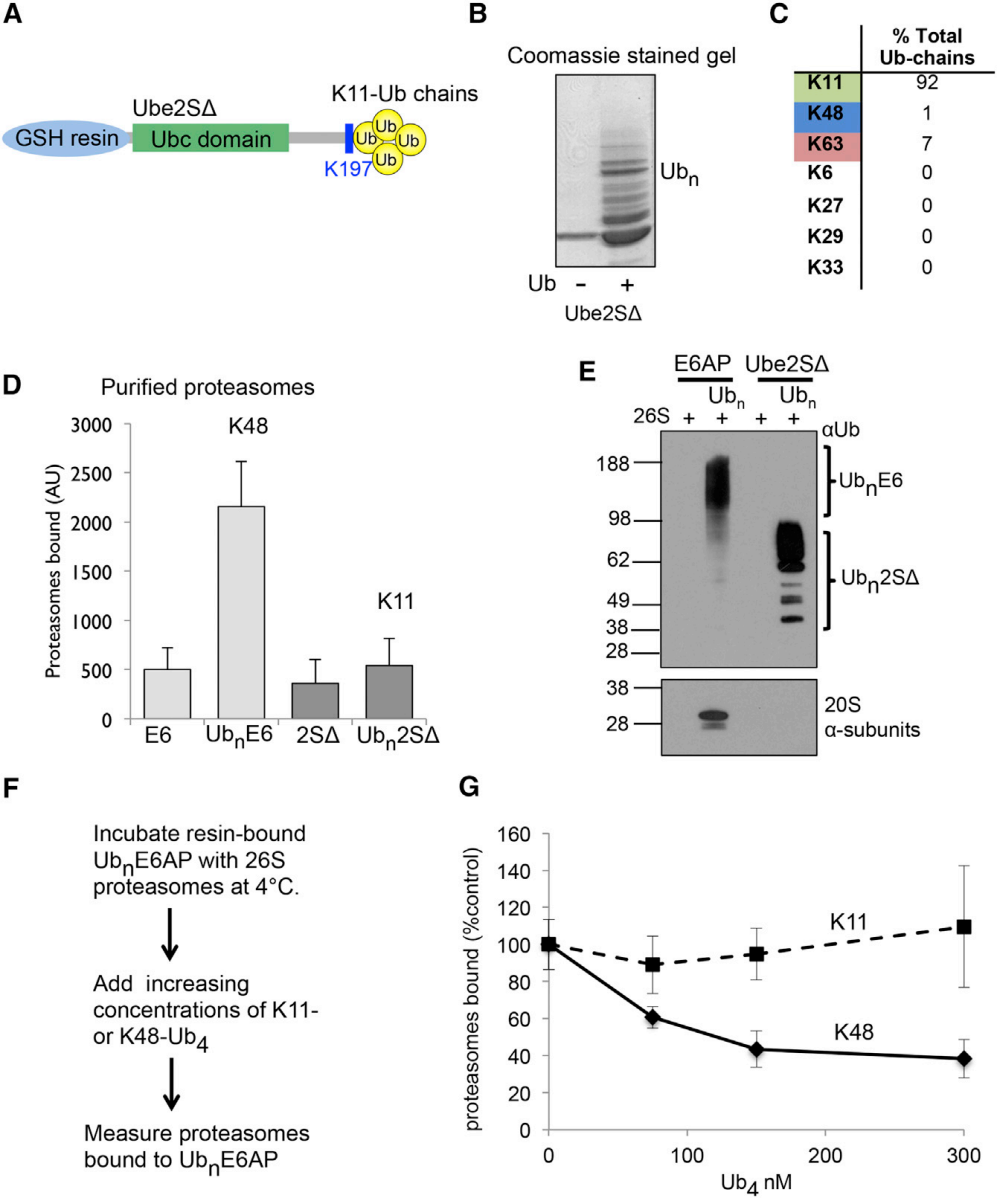
Homotypic K11-Linked PolyUb Chains Do Not Bind Strongly to the 26S Proteasome (A) Schematic shows truncated Ube2S, with the lysine rich C terminus removed, leaving a terminal lysine at position 197 (Ube2SΔ). (B and C) Homotypic K11-polyUb conjugates are formed on Ube2SΔ. Resin-bound Ube2SΔ was incubated with E1, Ub, AMSH, and ATP for 4 hr at 37°C. The resins were washed and either analyzed by SDS-PAGE and Coomassie staining (B) or the Ub linkages measured by AQUA MS (C). (D) K11-polyUb Ube2SΔ does not bind significantly to purified proteasomes. Polyubiquitinated E6AP and Ube2SΔ, or non-modified control resins, were incubated with purified 26S particles and the bound proteasomes were measured by LLVY-AMC cleavage. (E) Homotypic K11-polyUb chains do not bind to the proteasome. Polyubiquitinated E6AP and Ube2SΔ, or non-modified control resins, were incubated with purified 26S particles and the bound proteasomes were measured by immunoblot for the 20S α subunits. (F and G) K11-Ub_4_ does not compete with polyUb-E6AP to bind to the proteasome. Binding of 26S to PolyUb-E6AP in the presence of increasing concentrations of K48- or K11-Ub_4_ was measured. Binding of 26S proteasomes without the addition of Ub tetramers was taken as 100%. Values are the means ± SEM of three (D) or four replicates (G). E6, E6AP; 2S, Ube2SΔ; Ub_n_, polyUb chain of undefined length on the protein substrate.

To measure the binding of homotypic K11-polyUb chains to the proteasome, we generated K11-polyUb conjugate affinity columns by autoubiquitinating the E2 enzyme Ube2S, which forms polyUb chains on its lysine-rich C-terminal tail without an E3 ligase (Wickliffe et al., 2011; Wu et al., 2010). To prevent the formation of multiple monoUb linkages, we truncated the C terminus of Ube2S (Ube2SΔ), leaving only a terminal lysine at position 197 (K197) (Figure 1A), and we used mass spectrometry (MS) (Figure S1A) and Ub reactions containing methyl Ub (Ub^Me^) (Figure S1B) to confirm that K197 was the only lysine modified in Ube2SΔ. Truncations of Ube2S result in the formation of K63 linkages as well as K11 (Bremm et al., 2010). Therefore, the K63-specific deubiquitinating enzyme (DUB) AMSH was added to the autoubiquitination reactions to cleave any K63 linkages that occurred. Using this assay, we generated K11-polyUb linkages (Figure 1B) with 92% purity by MS and absolute quantification (AQUA) of Ub linkages (Figure 1C; Table S1).

We compared the binding of resin-bound K11- and K48-polyUb chains to purified mammalian proteasomes using a previously described assay (Nathan et al., 2013; Peth et al., 2010), which accurately measures the amount of 26S proteasomes bound to the Ub conjugates. K11-polyUb chains were formed on Ube2SΔ, and K48-polyUb chains formed by autoubiquitination of the HECT E3 ligase E6AP. The washed resin-bound K11- or K48-polyUb conjugates were incubated with pure 26S particles at 4°C, and the amounts of proteasomes bound were measured by cleavage of LLVY-AMC at 37°C. While K48-polyUb conjugates bound strongly to proteasome, the K11-polyUb chains did not bind 26S particles significantly (Figure 1D). The addition of AMSH to the Ube2SΔ Ub reaction did not block the binding of K11-polyUb chains to the proteasome, as K11-polyUb conjugates formed using a K11-only Ub mutant (Ub^K11^), without the addition of AMSH, also did not bind to proteasome (Figure S1C). To verify that proteasome activity correlated with amounts of 26S particles bound to the conjugates, we measured the levels of 20S α subunits by immunoblot. This confirmed that K11-polyUb chains did not bind to proteasome (Figure 1E).

The affinity of polyUb conjugates for the 19S is determined by Ub-chain length and the presence of an unfolded region within the protein substrate (Prakash et al., 2004). It was, therefore, possible that the length of the K11-polyUb chain on Ube2SΔ or the structure of Ube2S itself may account for the weak binding of the K11-polyUb conjugates to the proteasome. To exclude these possibilities, we used K11- and K48-polyUb chains of a fixed length and measured their ability to compete for Ub receptors on the 19S particle. As the attachment of four Ub molecules is sufficient to target proteins to the proteasome, we generated K11-linked tetraubiquitin (tetraUb) chains (K11-Ub_4_) using previously described methods (Dong et al., 2011). Coomassie staining and immunoblot confirmed that chains of predominantly four Ub molecules were formed, containing K11 linkages as determined by MS (Figures S1E–S1G). We next compared the binding of K11-Ub_4_ and commercially available K48-Ub_4_ to the proteasome, by their ability to compete with polyUb-E6AP for binding to 26S particles at 4°C (Figures 1F and 1G). Consistent with prior studies (Peth et al., 2010), the addition of unanchored K48-linked chains (300 nM) decreased polyUb-E6AP binding to proteasomes by 60%, with an approximate binding affinity constant (Ka) of 70 nM (Figure 1G). However, the addition of K11-Ub_4_ at concentrations up to 300 nM did not prevent polyUb-E6AP binding to 26S particles (Figure 1G). Thus, the proteasome does not bind K11-linked chains in the presence of K48-linked chains, consistent with our observation that K11-linked chains show only very weak association with the 26S proteasome.

### Proteasome Shuttling Factors Preferentially Bind Homotypic K48-PolyUb Conjugates Compared with K11-PolyUb Chains

**Figure 2:**
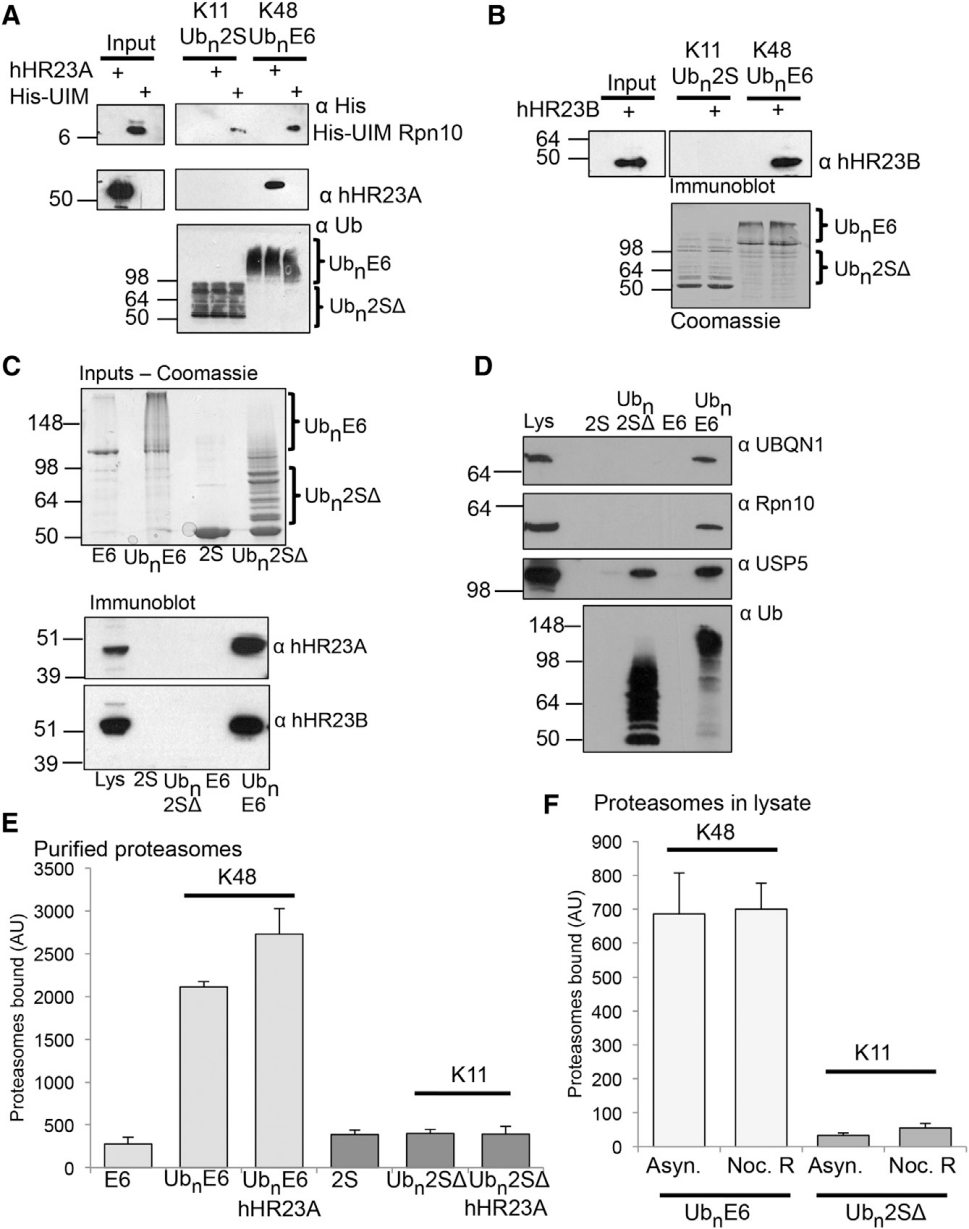
Proteasome-Shuttling Factors Preferentially Bind K48-PolyUb Conjugates Compared with K11-PolyUb Chains (A and B) Recombinantly expressed Rpn10, hHR23A, and hHR23B do not bind to polyUb-Ube2SΔ. PolyUb-E6AP and Ube2SΔ were incubated with 100 nM Rpn10-UIM, hHR23A (A), or hHR23B (B) for 30 min at 4°C, washed, and the bound proteins were visualized by immunoblot for His (Rpn10) or hHR23A/B. Ubiquitination of E6AP and Ube2SΔ was confirmed by immunoblot for Ub (A) or Coomassie (B). (C and D) Proteasome-shuttling factors in cell extracts do not bind homotypic K11-polyUb conjugates. PolyUb-E6AP and Ube2SΔ were incubated with HeLa cell extracts for 30 min at 4°C, washed, and the bound proteins were visualized by immunoblot for hHR23A and B (C) or Rpn10, UBQLN1, and USP5 (D). Ubiquitination of E6AP and Ube2SΔ was confirmed by Coomassie (C) or immunoblot for Ub (D). (E) HHR23A does not stimulate K11-polyUb conjugate binding to the 26S. Resin-bound polyUb-E6AP and Ube2SΔ were incubated with purified proteasomes or with 26S particles and 300 nM hHR23A. The bound proteasomes were measured by LLVY-AMC cleavage. (F) Proteasomes in lysates from asynchronous and mitotic exit cells bind to K48-polyUb chains, but not K11-PolyUb chains. Resin-bound PolyUb-E6AP and Ube2SΔ and non-modified controls were incubated with HeLa lysates (40 μg) from asynchronous cells (Asyn.) or cells synchronized and released from a nocodazole block (Noc. B) (Figure S2), for 30 min at 4°C, and the bound proteasomes were measured. Values are means ± SEM from three replicates.

Ubiquitinated proteins bind directly to the proteasome via Ub receptors on the 19S, but also can be delivered to the proteasome by shuttling factors, molecules that bind ubiquitinated proteins and facilitate their delivery to the 26S. We therefore examined whether the Rad23 proteins, hHR23A and hHR23B, and Rpn10 (a 19S Ub receptor that also is freely present in cells) bind to K11-polyUb chains. Recombinant forms of hHR23A, hHR23B, and the Ub-interacting motif (UIM) of Rpn10 were expressed, purified, and incubated with resin-bound polyUb-Ube2SΔ (K11 chains) and polyUb-E6AP (K48 chains). The fraction of proteins bound to the resins was determined by immunoblot (Figures 2A and 2B). The hHR23A and B (100 nM) bound only to the polyUb-E6AP and not to polyUb-Ube2SΔ (Figures 2A and 2B). The K48 conjugates also showed a marked preference (∼4-fold, ImageJ quantification) for the Rpn10 UIM compared with K11 chains (Figure 2A). The ability of proteasome-shuttling factors to selectively bind K48-polyUb compared with K11-polyUb conjugates also was confirmed with mammalian cell lysates. When resin-bound conjugates were incubated with HeLa cell extracts and the bound proteins visualized by SDS-PAGE, the Rad23 proteins (Figure 2C) and Rpn10 (Figure 2D) bound only the K48-polyUb chains. The selective binding of proteasome-shuttling factors for K48-polyUb chains also was observed for Ubiquilin 1 (UBQLN1/Dsk2). It is noteworthy that homotypic K11 conjugates do bind to non-linkage-selective ubiquitin-binding domains (UBDs), as polyUb-E6AP and Ube2SΔ bound the UBD containing protein USP5 similarly.

Proteasome-shuttling factors, such as the Rad23 proteins, not only bind K48-polyUb conjugates but also, at nanomolar concentrations, stimulate Ub conjugate binding to the proteasome (Nathan et al., 2013). To determine if Rad23 proteins facilitated the binding of K11 chains to the proteasome, we incubated resin-bound polyUb-Ube2SCΔ and polyUb-E6AP with 300 nM hHR23A and purified 26S proteasomes (Figure 2E). While hHR23A stimulated 26S binding to the K48 conjugates, it did not increase the affinity of K11 chains to the proteasome.

It remains possible that other proteins may facilitate delivery of K11-polyUb chains to the proteasome, particularly during exit from mitosis where K11-polyUb modifications have been associated with proteasomal degradation. To learn whether cells contain factors that might influence the binding of K11-polyUb chains to the 26S, resin-bound polyUb-Ube2SΔ and E6AP were incubated with asynchronous HeLa cell lysate or with synchronized cells released from nocodazole treatment (which induces mitotic exit; Figure S2) at 4°C, and proteasomes bound were measured by the cleavage of LLVY-AMC at 37°C (Figure 2F). After washing the resins, we found that proteasomes from asynchronous or mitotic exit lysates bound efficiently to the K48 chains, but not to K11 chains (Figure 2F), suggesting that other cellular factors do not increase the affinity of K11-polyUb chains for the proteasome. Thus, homotypic K11 chains are not a strong signal for proteasomal degradation.

### PolyUb Chains Containing Heterotypic K11 Linkages Can Bind to the 26S Proteasome

**Figure 3:**
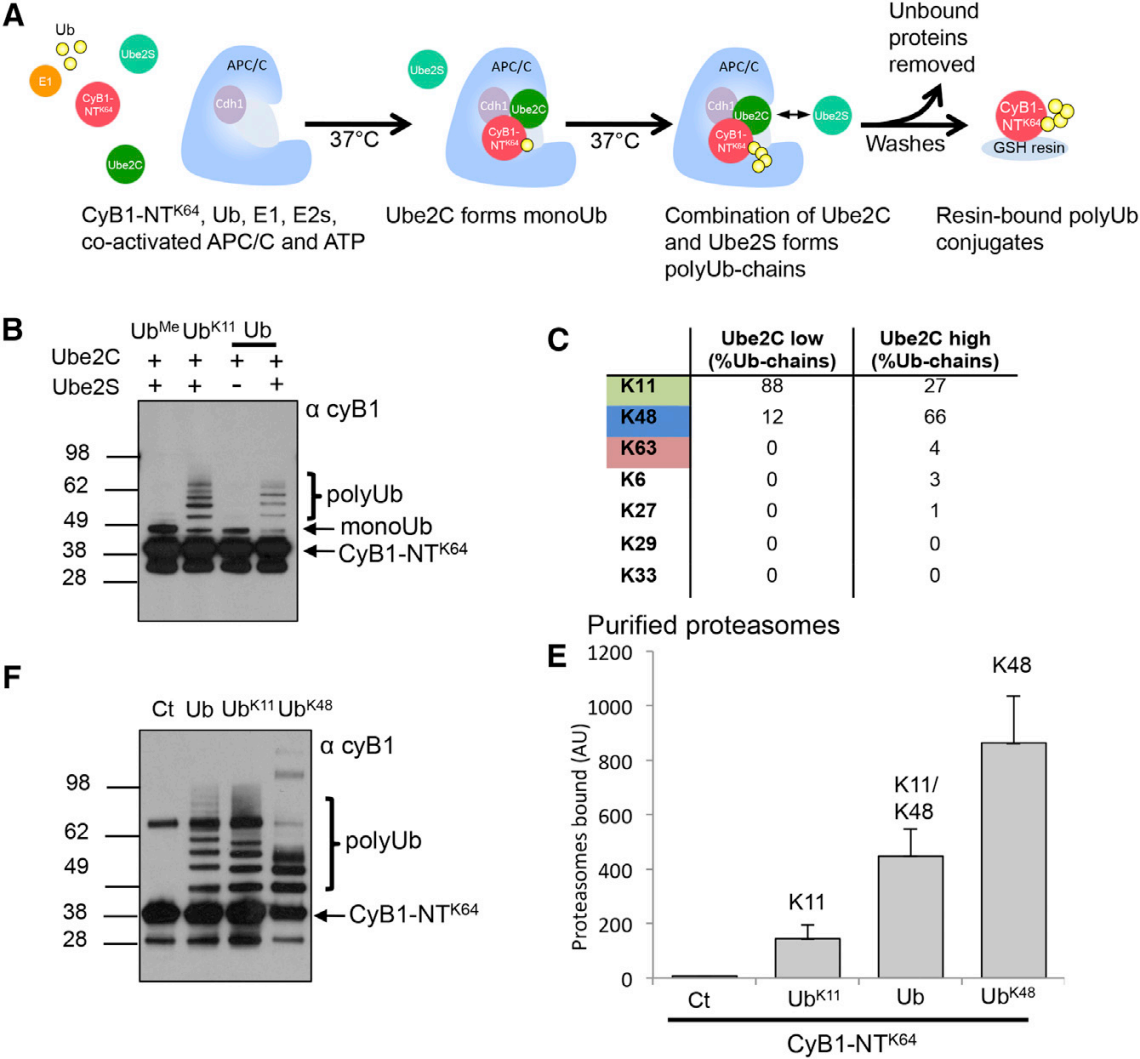
Heterotypic K11-PolyUb Chains Bind to the Proteasome (A–C) Synthesis of homotypic and heterotypic K11-polyUb chains on CyB1-NT^K64^ by the human APC/C. Resin-bound CyB1-NT^K64^ was incubated with co-activated APC/C, Ub (Ub, Ub^Me^, and Ub^K11^), and E2s (Ube2C and Ube2S) for 1 hr at 37°C, washed, and ubiquitination of CyB1-NT^K64^ was measured by immunoblot for cyclin B1 (B). Quantification of the Ub-linkages form was measured by MS (C). (D and E) Heterotypic, but not homotypic, K11-polyUb cyclin B1 binds to the proteasome. CyB1-NT^K64^ was ubiquitinated with Ub^K11^ and Ub^K48^ to form homotypic polyUb chains or ubiquitinated with wild-type Ub, forming K11/K48 heterotypic polyUb chains (D). These polyUb conjugates were incubated with purified proteasomes and the bound 26S particles were measured by LLVY-AMC cleavage (E). Values are the means ± SEM from three replicates.

To address whether heterotypic K11 linkages differ in their ability to bind to the proteasome compared with homotypic K11 chains, we developed an assay to form these different linkages on the cell-cycle regulator, cyclin B1, and measured their binding to purified 26S particles (Figure 3). Reconstituted co-activated APC/C (Zhang et al., 2013) was incubated at 37°C with a GST-bound N-terminal fragment of cyclin B1 (cyB1-NT), ATP, E1, and E2 enzymes to form K11-polyUb conjugates (Figure 3A). The unstructured N terminus of cyclin B1 contains 18 lysine residues close to the destruction box (D-box) and is ubiquitinated rapidly by co-activated APC/C. To prevent the formation of multiple monoUb linkages in the N-terminal fragment, we used the single-lysine construct at position 64 (cyB1-NT^K64^), which is still degraded by the proteasome in cell extracts (Dimova et al., 2012).

Two E2 enzymes are required to form polyUb chains on cyclin B1: (1) Ube2C (UbcH10), which adds the first Ub to the protein substrate but also can form polyUb chains; and (2) Ube2S, which elongates the chain with K11 linkages (Figure 3A). Ubiquitination assays containing Ub^Me^ or Ub with a single lysine at position 11 (Ub^K11^) confirmed that K64 is the only residue modified in the reaction (Figure 3B, lane 1) and that homotypic K11-polyUb chains were formed (Figure 3B, lane 2). By titrating the concentration of Ube2C, we were able to modify cyB1-NT^K64^ with a single Ub (Figure 3B, lane 3), or, alternatively, generate polyUb chains with the addition of Ube2S (Figure 3B, lane 4). High concentrations of Ube2C alone formed polyUb chains without the addition of Ube2S (data not shown). To determine the Ub linkages in these polyUb conjugates, we subjected the resin-bound polyUb cyB1-NT^K64^ to MS. Ubiquitination assays with 125 nM Ube2C and 900 nM Ube2S formed heterotypic chains containing predominantly K11 linkages but also K48, whereas 1 μM Ube2C formed heterotypic polyUb chains with multiple different lysine linkages (K6, K11, K27, K48, and K63) (Figure 3C; Table S2). Thus, co-activated APC/C can form K11/K48 heterotypic polyUb chains on cyclin B1, but the concentration of Ube2C is critical in determining the type of ubiquitin linkage formed.

To learn how homotypic and heterotypic K11-polyUb conjugates bind to the proteasome, we incubated the resin-bound chains with purified proteasomes. Homotypic K11- and K48-linked chains were formed using Ub with single lysines at position 11 or 48 (Ub^K11^ and Ub^K48^), while heterotypic K11/K48-linked chains were formed with 125 nM Ube2C and wild-type Ub (high concentrations of Ube2C were avoided to prevent the formation of multiple types of Ub linkage) (Figure 3D). Resin-bound K11-polyUb conjugates bound weakly to the proteasomes, whereas K48-polyUb conjugates bound strongly to the 26S (≈4.5-fold higher binding compared with K11 chains) (Figure 3E), consistent with our findings for polyUb-Ube2SΔ (Figure 1D). Heterotypic K11/K48-polyUb conjugates bound proteasomes with intermediate affinity compared with K11 and K48 homotypic chains (Figure 3E). The number of Ub molecules attached to cyB1-NT^K64^ did not account for the differences in conjugate binding to the proteasome, as both K11 homotypic and heterotypic chains were the same length. Interestingly, the homotypic K48-polyUb conjugates were shorter than K11-containing polyUb chains, but they bound most strongly to the proteasomes, consistent with K48 linkages being the predominant signal for proteasomal degradation. Thus, heterotypic chains containing K11 linkages can bind to the proteasome, but with lower affinity than K48-polyUb chains.

### Heterotypic K11-PolyUb Conjugates Facilitate Proteasomal Degradation Preferentially to Homotypic K11-PolyUb Chains

**Figure 4:**
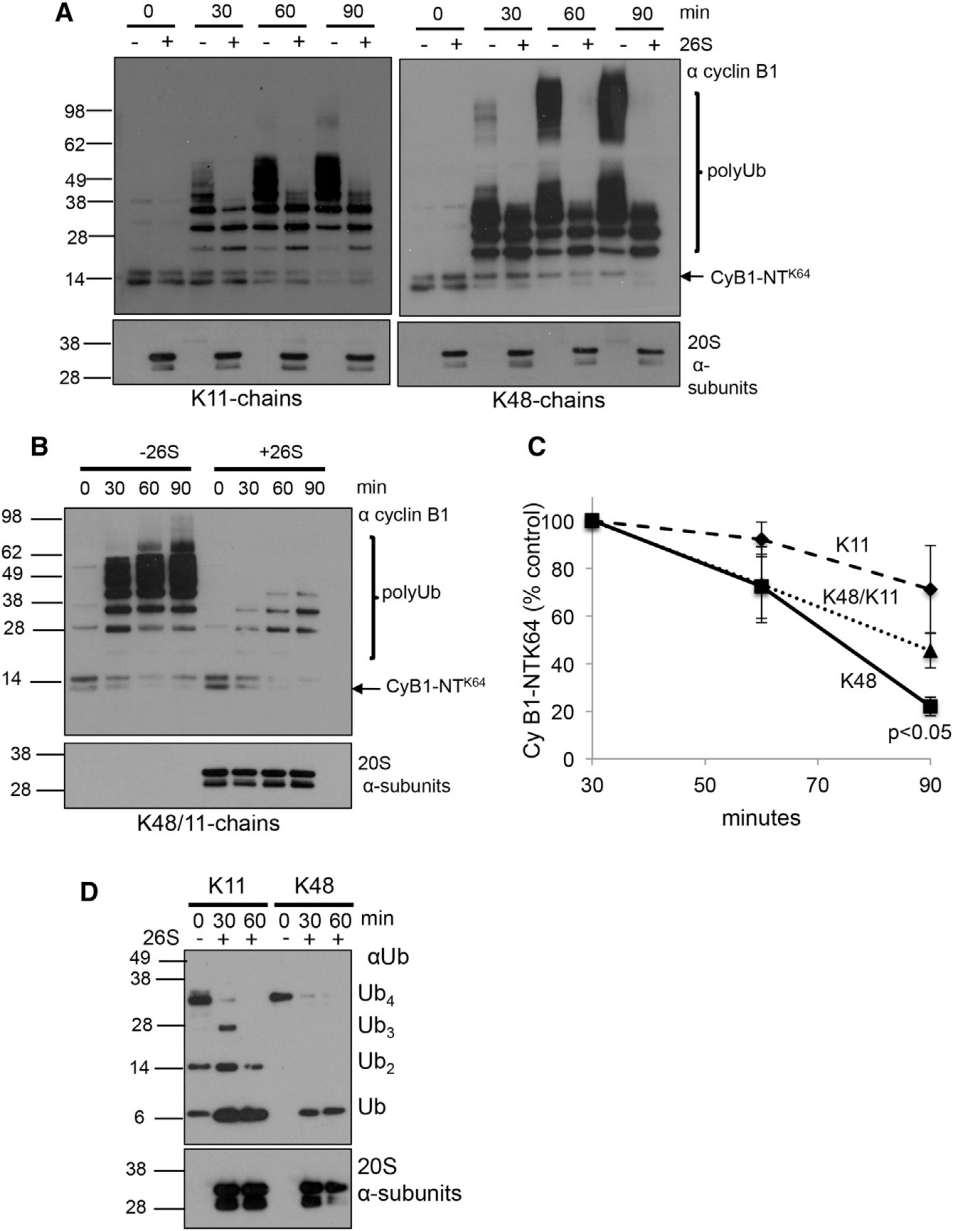
Homotypic K11-PolyUb Conjugates Are Disassembled, but Not Efficiently Degraded, by Mammalian Proteasomes (A–C) HA-tagged CyB1-NT^K64^ was incubated with co-activated APC/C, E1, E2s (Ube2C and Ube2S), and Ub, forming homotypic K11- and K48-polyUb chains (using Ub^K11^ and Ub^K48^) or heterotypic K11/K48-polyUb chains (using wild-type Ub). The 20 nM 26S proteasomes were added to the reactions and the samples were incubated at 37°C. The reactions were terminated by the addition of SDS loading buffer at 0, 30, 60, and 90 min, and ubiquitination and degradation of CyB1-NT^K64^ were measured by immunoblot for cyclin B1 (A). (C) Graphical presentation shows the means ± SEM of densitometric evaluation of the immunoblots from four separate experiments. Degradation of CyB1-NT^K64^ was measured from 30 min, allowing time for polyUb of cyclin B1 to occur during the first 30 min. Immunoblots for the 20S α subunits and the APC/C subunit cdc27 served as loading controls. (D) Homotypic K11- and K48-free polyUb chains are disassembled by proteasome-associated DUBs. The 150 nM K11-Ub_4_ and K48-Ub_4_ were incubated with 20 nM proteasomes at 37°C. The reactions were terminated by the addition of SDS loading buffer at 0, 30, and 60 min, and disassembly of the chains was visualized by immunoblot for Ub. Immunoblots for the 20S α subunits served as loading controls.

Proteasome-associated DUBs disassemble Ub chains and can regulate the rate of protein degradation by the 26S (Lee et al., 2010; Peth et al., 2009). As homotypic K11-polyUb conjugates did not bind strongly to the 26S, they should not be able to stimulate degradation by the proteasome; but, it remains unclear whether they can still be targeted by DUBs for disassembly. We therefore measured the disassembly and proteasomal degradation of CyB1-NT^K64^ modified with homotypic K11- and K48-polyUb chains or heterotypic chains containing K11/K48 linkages. HA-tagged CyB1-NT^K64^ was incubated with Ub (wild-type, Ub^K11^ or Ub^K48^) ATP, E1, E2 enzymes (Ube2C and Ube2S), co-activated APC/C, and purified 26S proteasomes at 37°C, and the ubiquitination and degradation of cyclin B1 was visualized by immunoblot (Figures 4A, 4B, and S2C). Incubation of all types of K11-polyubiquitinated CyB1-NT^K64^ with proteasomes resulted in shorter polyUb-chain lengths compared to ubiquitination reactions without the 26S. Three Ub molecules remained on the K11 and K48 homotypic chains, while four Ub molecules were visualized on the heterotypic chains (Figures 4A and 4B). Complete disassembly of the polyUb chains presumably did not occur as cyclin B1 can be continually re-ubiquitinated in the assay. To confirm that the proteasome-associated DUBs can disassemble K11 linkages, homotypic K11-Ub_4_ and K48-Ub_4_ were incubated with purified 26S proteasomes and the disassembly of the chains was visualized by immunoblot (Figure 4D). The 26S proteasomes disassembled free homotypic K11-Ub_4_, but at a slower rate than K48-Ub_4_ (Figure 4D).

The degradation of polyUb-CyB1-NT^K64^ was quantified by densitometry of the immunoblots between 30 and 90 min (Figure 4C), as it takes approximately 30 min to form polyUb conjugates of sufficient length to stimulate proteasomal degradation. After 60 min, both homotypic K48- and heterotypic K11/K48-polyUb CyB1-NT^K64^ levels were decreased by ∼25%, but there was no change in K11-polyUb CyB1-NT^K64^ levels. At 90 min, nearly 80% of K48-polyUb CyB1-NT^K64^ was degraded, whereas heterotypic K11/48-polyUb conjugates were decreased by ∼50% (Figure 4C). K11-polyUb CyB1-NT^K64^ decreased minimally over 90 min (Figure 4C), confirming that homotypic K11-polyUb conjugates do not stimulate proteolysis by the 26S. Thus, while proteasome-associated DUBs may disassemble polyUb chains containing homotypic or heterotypic K11 linkages, they do not degrade homotypic K11-polyUb conjugates. This also implies that polyUb chains that do not bind significantly with the 19S can still be targeted for disassembly by proteasome-associated DUBs.

## Discussion

Here we have shown that the proteasome can distinguish between K11-linked ubiquitin chains of distinct topology, and we have identified that homotypic K11-polyUb conjugates do not bind strongly to pure mammalian proteasomes. We find that homotypic K11-polyUb chains do not bind with sufficient affinity or avidity to the 26S to stimulate degradation of the protein substrate, and that there are clear differences in the affinity of K11- and K48-linked chains for the 26S. By directly examining the binding of K11 conjugates to the proteasome, we found that Ub receptors on the 19S can select between a homotypic versus heterotypic chain structure. This has important implications for the mechanisms by which atypical Ub chains are recognized and their functions diversified.

### The Topology of Homotypic K11-PolyUb Chains May Prevent Their Association with the Proteasome Ub Receptors

K11-Ub dimers adopt a conformation distinct from other forms of Ub linkages, but the exact topology is unclear, as the Ub-to-Ub orientations differ markedly between the two known crystal structures (Bremm et al., 2010; Matsumoto et al., 2010). In particular, the hydrophobic isoleucine (I44) region, which forms the main binding surface of Ub, is exposed in one model and buried in the other. Furthermore, neither crystal structure fully agrees with the nuclear magnetic resonance (NMR) structure (Castañeda et al., 2013). Our findings show that long homotypic K11-polyUb chains must adopt a conformation distinct from both K48 and K63 chains, as these both bind the proteasome in vitro, and suggest that the main binding surface for Ub 19S receptors, the I44 region, is not exposed in K11-polyUb conjugates. Indeed, the solution NMR data show an interaction of the distal Ub I44 region with the proximal Ub, forming a structurally distinct Ub-binding surface in K11-Ub dimers (Castañeda et al., 2013).

It has been reported that K11-polyUb chains formed by the APC/C bind to hHR23B and S5A/Rpn10 to facilitate the degradation of cell-cycle regulators (Jin et al., 2008; Meyer and Rape, 2014), but ambiguity remains regarding which chain types mediated these effects. A key advance of our study is that we used polyUb conjugates formed on a single-lysine residue, thereby preventing the formation of multiple monoUb linkages, which are sufficient to degrade cyclin B1 in *Xenopus* cell extracts (Dimova et al., 2012). Whether multiple homotypic K11 chains on the same substrate signal proteasomal degradation is unclear, but recently Lu et al. (2015) demonstrated that multiple short homotypic K48 chains on the cell-cycle substrate Securin are more efficient than homotypic K11 chains in stimulating proteasome degradation. In addition, our use of reconstituted co-activated human APC/C avoided contaminating factors that may have been present in prior assays, which typically used immunoprecipitation to isolate the APC/C. Although unknown cellular factors may facilitate the binding of homotypic K11-polyUb chains to the proteasome, this is unlikely as proteasomes in asynchronous and nocodazole-released cell extracts did not bind to homotypic K11-polyUb conjugates (Figure 2).

Our findings extend earlier conclusions that Rad23 proteins bind selectively to K48-polyUb chains, as both full-length hHR23A and B did not bind K11-polyUb conjugates. Prior studies, using the isolated second UBA domain of hHR23A, showed that K11-Ub dimers bound with approximately ten times lower affinity than K48 dimers (≈160 μM compared to ≈18 μM) (Castañeda et al., 2013), consistent with our findings that the Rad23 proteins are not UBPs for homotypic K11 chains.

### Heterotypic K11-PolyUb Conjugates Adopt Conformations that Allow Binding to the Proteasome

Even though the main linkage in the heterotypic polyUb chains was through K11, the formation of small percentages of K48 linkages allowed conjugates to bind to the proteasome in vitro. This suggests that heterotypic polyUb chains comprised of predominantly K11 linkages adopt conformations that can bind to the 19S, presumably by exposing the I44 Ub-binding surface, or alternatively that the presence of the K48 linkages permit association with the 26S ubiquitin receptors. While it is beyond current techniques to determine the exact Ub conformations in these linkages, it would be of interest to learn how different types of mixed K11-Ub chains bind proteasome Ub receptors. Our findings, however, are consistent with other studies showing that K11/K48-branched polyUb conjugates can act as proteasomal signals (Meyer and Rape, 2014).

The formation of heterotypic chains with multiple different lysine linkages on cyclin B1 was determined by the concentration of Ube2C within the APC/C Ub reaction. Low concentrations of Ube2C formed heterotypic chains predominantly containing K11/48 linkages, but at high concentrations Ube2C formed complex chains with six different Ub linkages. This ability of the APC/C to form multiple Ub linkages also has been observed in *Xenopus* extracts, where APC/C in combination with high concentrations of the E2 UBC4 (up to 4 μM) formed polyUb chains on cyclin B1 containing K11, K48, and K63 linkages (Kirkpatrick et al., 2006). Whether this degree of complex Ub-chain formation is physiologically relevant, or simply due to the high concentrations of E2s used in the in vitro assays, remains to be determined. However, we avoided the use of micromolar concentrations of Ube2C, as prior studies showed that complex heterotypic polyUb chains are prevented from forming in cells due to association with Rpn10 (Kim et al., 2009).

The removal of the Ub molecules by proteasome-associated DUBs can regulate the rate of proteolysis. Both homotypic and heterotypic K11-polyUb conjugates were disassembled by the proteasome-associated DUBs, but only K11/K48-polyUb conjugates were degraded. This suggests that binding of polyUb conjugates to high-affinity 19S receptors is not required to activate the proteasome-associated DUBs for the disassembly of homotypic K11 chains. It is noteworthy that heterotypic K11-polyUb conjugates were disassembled to four Ub moities on CyB1-NT^K64^, whereas homotypic K48 and K11 chains were disassembled to three Ub molecules. The reason for this difference in DUB activity between heterotypic and homotypic chains is not clear, but our findings are consistent with studies that show disassembly of homotypic and heterotypic free K11 chains by proteasomes (Mansour et al., 2015). This also implies that the DUBs associated with the proteasome can modify Ub linkages on proteins despite their lack of significant association with the high-affinity Ub proteasome receptors and without their subsequent degradation.

### What Are the Roles of Homotypic K11 Linkages in Cells?

Our study provides a mechanistic basis for prior unexplained observations that K11-Ub linkages are involved in proteasome-independent pathways, including TNF-α signaling (Dynek et al., 2010), endocytosis of plasma membrane proteins (Boname et al., 2010), and hypoxia signaling (Bremm et al., 2014). In particular, the ability of the K11-selective DUB, Cezanne, to regulate hypoxia-inducible factor (HIF)α in a proteasome-independent manner is consistent with our findings that homotypic K11-polyUb does not bind to the 26S.

An intriguing possibility is that K11-Ub linkages may in fact stabilize proteins and prevent their degradation. Two studies support this hypothesis as follows: (1) K11 linkages formed on β-catenin by the Fanconi Anaemia Ub ligase prevent its degradation (Dao et al., 2012); and (2) the E3 ligase RNF26 forms K11 linkages on a mediator of viral interferon induction (MITA), stabilizing the protein and regulating the innate immune response (Qin et al., 2014). Whether the ability of K11-polyUb linkages to stabilize proteins is due to the topology of the chains, which may prevent the I44 Ub-binding surface from being exposed, or recognition of K11 linkages by as yet unknown K11-selective binding proteins remains to be determined. Importantly, these in vivo studies did not determine the nature of K11 linkages formed on proteins. Our findings that the proteasome can distinguish between homotypic and heterotypic K11-polyUb chains may now explain why K11 conjugates can result in both protein stabilization and proteasomal degradation. This also may highlight a broader role for chain topology in the selection of polyUb-conjugated proteins by UBPs, thereby determining their cellular fate.

## Experimental Procedures

### Plasmids, Antibodies, and Protein Expression

A complete list of plasmids and antibodies is available in the Supplemental Experimental Procedures. The E1, E2, and E3 enzymes and cyB1-NT^K64^ (GST, His, or HA tagged) were expressed in *E. coli* and purified using standard techniques (full details are in the Supplemental Experimental Procedures). Rad23 proteins and the UIM of Rpn10 were expressed and purified as before (Nathan et al., 2013). Recombinant apo human APC/C was expressed in insect cells co-infected with baculovirus and purified as described previously (Zhang et al., 2013).

### Ubiquitination Assays and Purification of 26S Proteasomes

PolyUb-E6AP was formed as before (Nathan et al., 2013). Resin-bound Ube2SΔ was ubiquitinated using 50 nM E1, 4 mM ATP, and 50 μM Ub. After autoubiquitination, the GST-bound Ub conjugates were washed five times with 50 mM Tris-HCl (pH 7.5), 150 mM NaCl, and 1 mM DTT. K11-Ub_4_ was synthesized using Ube2S as described previously (Bremm et al., 2010). Ub linkages formed on Ube2SΔ were measured by MS (details in the Supplemental Experimental Procedures).

The 5 μM GST-tagged cyB1-NT^K64^ was ubiquitinated using a reaction containing the following: 40 mM Tris (pH 7.4), 10 mM MgCl_2_, 0.6 mM DTT, 250 nM E1, 125 nM−1.25 μM Ube2C, 900 nM Ube2S, 40 nM APC/C, 2 μM Cdh1, 50 μM Ub, 5 mM ATP, and 0.25 mg/ml BSA. Reaction mixtures were incubated at 37°C for up to 1 hr. The ubiquitinated GST-cyB1-NT^K64^ was then bound to glutathione resin, washed, and analyzed by SDS-PAGE and western blotting. Ub linkages formed on cyB1-NT^K64^ were measured by MS (details in the Supplemental Experimental Procedures).

The 26S particles were purified from rabbit muscle by the Ubl-affinity method in the presence of 150 mM NaCl, as previously described (Besche et al., 2009).

### Measurements of Proteasome Binding and Degradation

The 26S proteasomes (10 nM) were incubated with resin-bound polyUb conjugates (≈20 nM), and proteasome activity was measured by cleavage of suc-LLVY-AMC, as described previously (Nathan et al., 2013; Peth et al., 2010). For proteasome-binding experiments using cell extracts, HeLa cells were lysed in 25 mM HEPES (pH 7.5), 150 mM NaCl, 5 mM MgCl2, 1 mM DTT, 1 mM ATP, and 10% glycerol.

To measure the proteasomal degradation of cyclin B1, 5 μM HA-tagged CyB1-NT^K64^ was ubiquitinated using co-activated APC/C as described, except that 20 nM 26S particles were added to the reaction mixture. Samples were incubated at 37°C for 0, 30, 60, and 90 min, and reactions were terminated by the addition of SDS-PAGE loading buffer. The degradation of CyB1-NT^K64^ was determined by densitrometric analyses of the immunblots for cyclin B1 (ImageJ software).

## Author Contributions

G.L.G., I.T.L., and J.A.N. designed the studies and performed the experiments. G.L.G. and J.A.N. wrote the manuscript. M.P.W., S.P.G., and R.A. performed the MS analyses.

## Supplemental Information

Document S1. Supplemental Experimental Procedures and Figures S1–S3

Table S1. MS Quantification of Ubiquitinated Ube2SK1, Related to Figure 1

Table S2. MS Quantification of Ubiquitinated CyB1-NT^K64^, Related to Figure 3

Document S2. Article plus Supplemental Information
