# Supplementary material for: The Proteasome Distinguishes between Heterotypic and Homotypic Lysine-11-Linked Polyubiquitin Chains
Source: Cell Rep. 2015 Jul 16;12(4):545–53. doi: 10.1016/j.celrep.2015.06.061 (PMC4533228; doi:10.1016/j.celrep.2015.06.061)
Supplement: Document S1. Supplemental Experimental Procedures and Figures S1–S3 [file mmc1.pdf]

Cell Reports
Supplemental Information

The Proteasome Distinguishes between Heterotypic and Homotypic Lysine-11-Linked Polyubiquitin Chains

Guinevere L. Grice, Ian T. Lobb, Michael P. Weekes, Steven P. Gygi, Robin Antrobus, and James A. Nathan

SUPPLEMENTARY INFORMATION

Figure S1: Synthesis of K11-polyUb conjugates on Ube2S and free K11-Ub$_4$ (related to Figure 1).

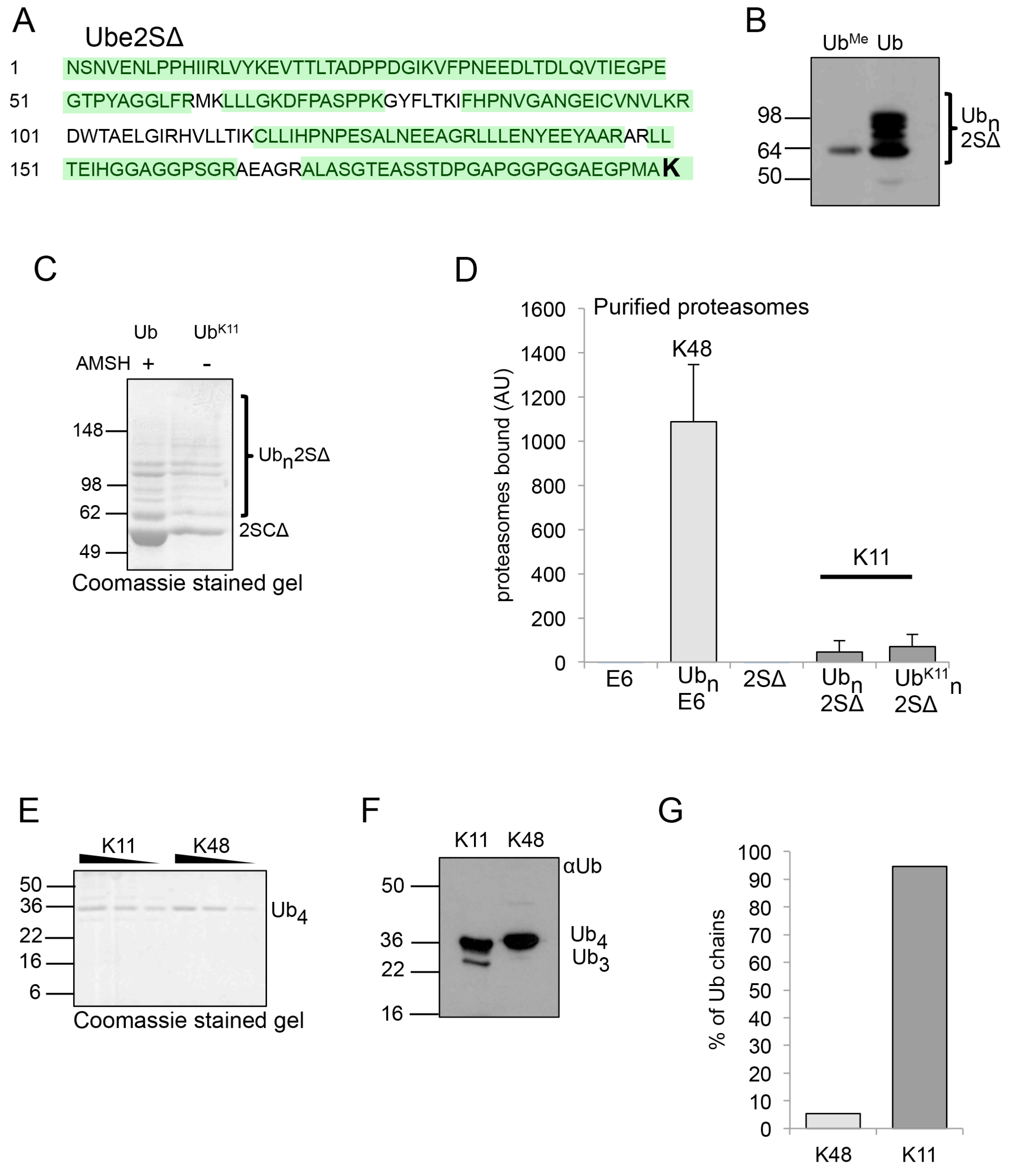

(A, B) Ube2SΔ forms polyUb chains on a single lysine at position 197. Resin-bound Ube2SΔ was ubiquitinated using 50nM E1, 4mM ATP and 50μM Ub (wildtype of Ub^{Me}) for 4 hr at 37°C. After washing, the resin-bound conjugates were analysed by MS (A) or by western blotting for Ub (B). Green represents the peptide coverage by

MS (A). The highlighted lysine at position 197 was the only residue modified by ubiquitin (A).

(C, D) K11-polyUb Ube2SΔ does not bind significantly to purified proteasomes. Ube2SΔ was ubiquitinated with wildtype Ub or Ub^{K11} as described. AMSH was only added to the wildtype Ub reactions. Polyubiquitination of Ube2SΔ was visualised by Coomassie (C). The polyubiquitinated E6AP and Ube2SΔ, or non-modified control resins, were incubated with purified 26S particles and the bound proteasomes were measured by LLVY-AMC cleavage (D).

(E-G) Purity of K11-Ub_4. K11-Ub_4 was generated as described (see supplementary experimental procedures), and the purity of the samples was measured by Coomassie (E) and immunoblot for Ub (F). Ub-linkages were analysed by MS using the precursor ion intensities (G).

Figure S2: Validation of nocodazole released mitotic cell extracts (related to Figure 2).

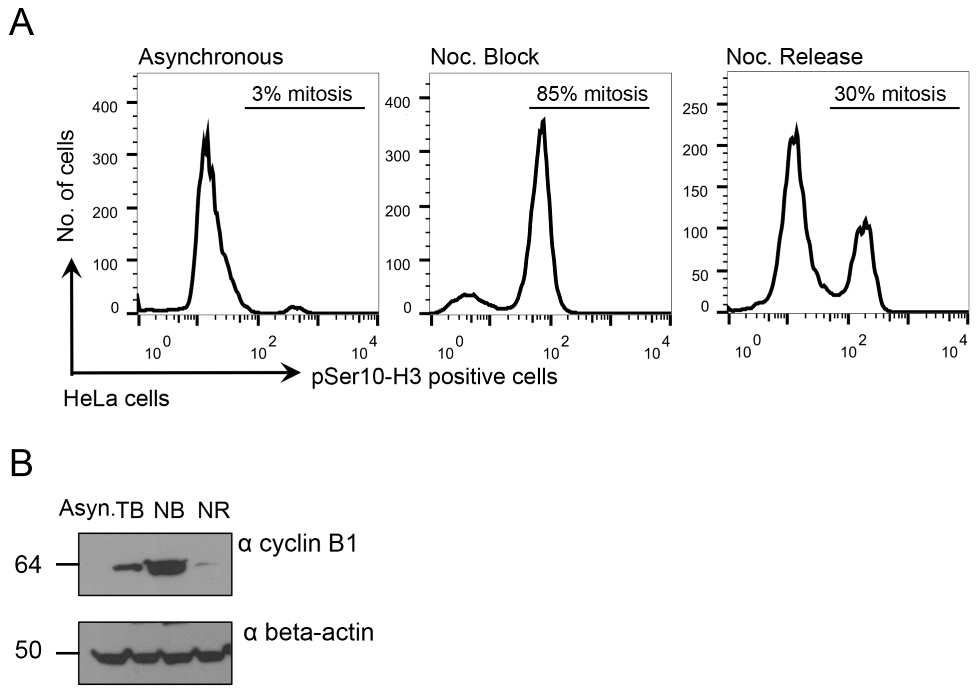

(A) Thymidine synchronised HeLa cells accumulate in mitosis following nocodazole treatment. HeLa cells were cultured in the presence of 2 mM thymidine for 24 hr before release into fresh media for 4 hr and subsequent 11 hr incubation with 0.05 ng/ml nocodazole. Cells were then released into fresh media for 2 hr. Mitotic cells were estimated by intracellular flow cytometry for Serine 10 phosphorylation on Histone H3 (pSer10-H3) in asynchronous, nocodazole blocked and nocodazole released cells (% cells in mitosis).

(B) Cyclin B1 is rapidly degraded following release from a nocodazole block. 1x 10^5 cells were lysed in SB buffer (20 mM HEPES pH 7.5, 1.5 mM $MgCl_2$, 1 mM DTT, 5 mM KCl, PMSF and Roche PI cocktail) before homogenisation by two freeze-thaw cycles and passage through a needle. After a 30 min centrifugation at 17,000 *g*, SDS loading buffer was added and the samples subjected to SDS-PAGE and immunoblot for cyclin B1. β-actin served as a loading control.

Asyn.=asynchronous, TB=thymidine block, NB=nocodazole block, NR=nocodazole release.

Figure S3: Heterotypic but not homotypic K11-polyUb conjugates facilitate proteasomal degradation (related to Figure 4).

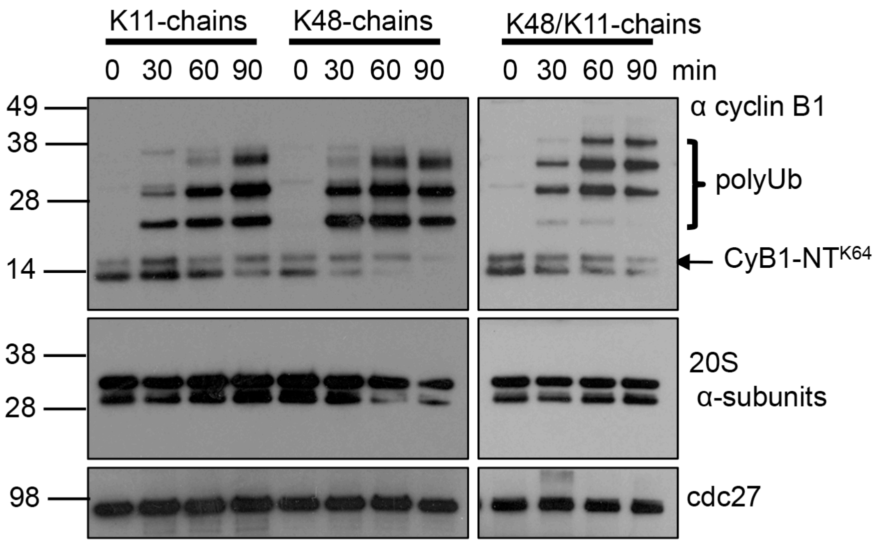

Proteasome associated DUBs disassemble heterotypic and homotypic K11-linked polyUb chains. HA-tagged CyB1-NT^{K64} was incubated with co-activated APC/C, E1, E2s (Ube2C and Ube2S) and Ub, forming homotypic K11- and K48-polyUb chains (using Ub^{K11} and Ub^{K48}) or heterotypic K11/K48-polyUb chains (using wildtype Ub). 20nM 26S proteasomes were added to the reactions and the samples incubated at 37°C. The reactions were terminated by the addition of SDS loading buffer at 0, 30, 60 and 90 min. Ubiquitination and disassembly of polyUb chains was measured by immunoblot for cyclin B1.

Supplementary File 1. Mass spectrometry quantification of ubiquitinated Ube2SK1 (related to Figure 1).

Supplementary File 2. Mass spectrometry quantification of ubiquitinated CyB1-NT^{K64} (related to Figure 3).

EXPERIMENTAL PROCEDURES

Plasmids, reagents and antibodies

His-Ube2S was a gift from Marc Kirschner (Harvard Medical School). GST-Ube2SΔ was formed by cloning DNA encoding residues 1 to 196 of Ube2S into the bacterial expression vector pGEX6P1, using the following primers: Ube2S BamH1 For CGGGA TCCAACTCCAACGTGGAGAACC and Ube2S Not1 Rev TTGCGGCCGCCTACTTGGCCATGGGACCCTCA. pET28a HA-cyB1-NT^{K64}-His was a gift from Randall King (Harvard Medical School). GST-cyB1-NT^{K64}-His was formed by cloning cyB1-NT^{K64} into pGEX6P1 using the primers: CyB1 BamHI For CGGGATCCGGCACCATGGCGCTCCGAGTCACGC and cyB1 NotI Rev TTGCGGCCGCCTAGGGAGCGTGATGGTGATG. GST-AMSH (pGEX6P1-AMSH) was a gift from Sylvie Urbé (University of Liverpool). Ubiquitins (Ub, Ub^{Me}, Ub^{K11} and Ub^{K48}) and Ube2C were purchased from Boston Biochem.

The following antibodies were used: mouse monoclonal to Ub (P4D1, Santa Cruz), rabbit polyclonal to hHR23A (Bethyl Laboratories), rabbit polyclonal to hHR23B (Bethyl Laboratories), rabbit polyclonal to His tag (ab9108, Abcam), mouse monoclonal to proteasome 20S α subunits (MCP231, Enzo Life Sciences), mouse monoclonal to cyclin B1 (BD Pharmingen), mouse monoclonal to cdc27 (AF3.1, Santa Cruz), rabbit polyclonal to USP5 (Bethyl Laboratories), mouse monoclonal to Rpn10 (S5a-18, Enzo Life Sciences) and rabbit monoclonal to UBQLN1 (Cell Signaling).

Protein expression and purification

Human E1, His-Ubch5b, GST-E6AP, hHR23A and hHR23B were expressed and purified as described previously (Nathan et al., 2013). GST conjugated Ube2SΔ, AMSH and cyB1-NT^{K64} were expressed in BL21DE3star *E. Coli*, and purified using glutathione sepharose (GE Healthcare). For AMSH, the GST tag was cleaved, using GST tagged Prescission protease (GE Healthcare), and removed by incubation with

the glutathione sepharose. His-tagged proteins were similarly expressed in *E. Coli* and purified using a NiNTA column and FPLC.

K11-Ub_4 were synthesised as described by Bremm and Komander, 2012, with some modifications. 4.8μM GST-Ube2SΔ or GST-Ube2SUBD were incubated with 250nM E1, 3mM Ub, 10mM ATP, 400nM AMSH in conjugation buffer (150mM NaCl, 50mM Tris pH 7.4, 1mM DTT, 1mM $MgCl_2$) for 18hr at 37°C. The samples were incubated with 60mM DTT at 4°C for 10 min and then 50mM NH_4Ac was added to precipitate all proteins apart from Ub, and incubated at 4°C for 30 min. Precipitates were removed by passing through a 0.2μm filter and the Ub chains separated by cation exchange chromatography using a Mono-S 5/50 GL column (GE Healthcare). Pooled Ub_4 fractions were dialysed against 20mM Tris pH 7.4, 1mM DTT, and the Ub-chains concentrated using a 3000MWCO Amicon centrifugal concentrator (Millipore).

Mass spectrometry

Proteins were resolved using a 4-12% pre-cast polyacrylamide gel. Lanes were cut into equal size chunks with proteins reduced, alkylated and digested in-gel. Tryptic peptides were analysed by LC-MSMS using a Q Exactive coupled to an RSLCnano 3000. Peptides were resolved and sprayed using a 50cm EASY spray column with MSMS acquired by top 6 DDA. Data was processed in Proteome Discoverer 1.4 using the Sequest search engine. Searches were performed against a Uniprot human database (20,176 entries, downloaded 03/06/14) with Cam C as a fixed modification, oxidised M, GlyGly K, deamidated N/Q as variable modifications and a maximum of 2 missed cleavages. Peak area detection was enabled with peptides reporting average area of the three most abundant of each detected species. Peptides were filtered to 0.01 FDR and identified branch sites were expressed as a percentage of the summed intensity of all quantified ubiquitin peptides.

AQUA mass spectrometry

The polyubiquitinated Ube2SΔ gel piece was sliced into 3 parts and each slice was destained and dehydrated in Acetonitrile. Proteins were then digested in-gel with Trypsin for 3 hr. Ubiquitin AQUA analysis was performed as previously described(Kirkpatrick et al., 2006). Briefly, peptides were eluted, and peptide amount estimated. AQUA peptides were mixed at 600fmol per microgram Ube2SΔ peptide, and the entire samples were analysed on a Q Exactive mass spectrometer equipped with an Agilent 1100 binary pump and a Famos microautosampler. Peptides were separated using a gradient of 6 to 28% Acetonitrile in 0.125% Formic acid over 120 minutes. Peptides were detected in the Orbitrap by means of a data-dependent top-20 method. Extracted ion chromatograms were generated using Xcalibur v2.2 software (Thermo). Chromatographic coelution of heavy and light peptide pairs and accurate peak integration were manually confirmed. The abundance of each peptide was determined by taking the ratio of the integrated areas for the light sample peptide versus the heavy AQUA internal standard peptide (Kirkpatrick et al., 2006). The percentage K11-linkages was calculated as (fmol K11) / (fmol K6 + K11 + K27 + K29 + K33 + K48 + K63).

Reference

Kirkpatrick, D.S., Hathaway, N.A., Hanna, J., Elsasser, S., Rush, J., Finley, D., King, R.W., and Gygi, S.P. (2006). Quantitative analysis of in vitro ubiquitinated cyclin B1 reveals complex chain topology. Nature cell biology *8*, 700-710.
